# Ectomycorrhizal Influence on the Dynamics of Sesquiterpene Release by *Tricholoma vaccinum*

**DOI:** 10.3390/jof8060555

**Published:** 2022-05-24

**Authors:** Marycolette Ndidi Ezediokpu, Katrin Krause, Maritta Kunert, Dirk Hoffmeister, Wilhelm Boland, Erika Kothe

**Affiliations:** 1 Institute of Microbiology, Microbial Communication, Friedrich Schiller University Jena, Neugasse 25, 07743 Jena, Germany; marycolette.ndidi.ezediokpu@uni-jena.de (M.N.E.); katrin.krause@uni-jena.de (K.K.); 2 Max Planck Institute for Chemical Ecology, Bioorganic Chemistry, Hans-Knöll-Straße 8, 07745 Jena, Germany; mkunert@ice.mpg.de (M.K.); boland@ice.mpg.de (W.B.); 3 Department of Pharmaceutical Microbiology, Hans Knöll Institute, Friedrich Schiller University Jena, Winzerlaer Strasse 2, 07745 Jena, Germany; dirk.hoffmeister@hki-jena.de

**Keywords:** *Tricholoma vaccinum*, ectomycorrhiza, volatilome, sesquiterpene synthases

## Abstract

*Tricholoma vaccinum* is an ectomycorrhizal basidiomycete with high host specificity. The slow-growing fungus is able to produce twenty sesquiterpenes, including α-barbatene, sativene, isocaryophyllene, α-cuprenene, β-cedrene, ß-copaene, 4-epi-α-acoradiene, and chamigrene in axenic culture. For the three major compounds, Δ^6^-protoilludene, β-barbatene, and an unidentified oxygenated sesquiterpene (*m/z* 218.18), changed production during co-cultivation with the ectomycorrhizal partner tree, *Picea abies*, could be shown with distinct dynamics. During the mycorrhizal growth of *T. vaccinum*–*P. abies*, Δ^6^-protoilludene and the oxygenated sesquiterpene appeared at similar times, which warranted further studies of potential biosynthesis genes. In silico analyses identified a putative protoilludene synthesis gene, *pie1*, as being up-regulated in the mycorrhizal stage, in addition to the previously identified, co-regulated geosmin synthase, *ges1*. We therefore hypothesize that the sesquiterpene synthase *pie1* has an important role during mycorrhization, through Δ^6^-protoilludene and/or its accompanied oxygenated sesquiterpene production.

## 1. Introduction

Basidiomycete, mushroom-forming fungi are well-known producers of sesquiterpenoids [1,2]. This class of natural products includes well-known plant compounds, e.g., zingiberene, from the spice and traditional medicinal plant, ginger (*Zingiber officinale*), or the sesquiterpene lactones such as the anti-malaria compound artemisinin [1]. After backbone assembly, sesquiterpenoids undergo modifications to form, e.g., the fresh soil odor compound geosmin or many essential oils [1]. Aside from plants, volatiles including sesquiterpenes are prevalent in fungi, where the mushroom odor compounds 3-octanone and 1-octen-3-ol are found in addition to merosesquiterpenoids, the latter being formed from precursors with mixed origin in biosynthesis through both the mevalonate and non-mevalonate pathways [3]. A wealth of compounds was isolated and identified, e.g., from plant endophytic fungi [4,5]. Sesquiterpenes from fungi play a significant role in their headspace chemistry [6], and just like other volatile organic compounds (VOCs), they are regarded as infochemicals that activate inter- and intraspecies communication [2,7,8]. Their potential functions include interactions with other organisms, from immunomodulatory/pathogenicity-related functions, through interspecific interactions, to mutual symbiosis [9,10]. Production might be triggered by environmental, as well as developmental cues [11,12,13]. Hence, the function of sesquiterpene volatiles in mutually symbiotic interactions was addressed to unravel the role of these compounds as natural communication molecules in these co-evolved systems.

*Tricholoma vaccinum* is a late-stage ectomycorrhizal fungus, characterized by slow growth and high host specificity towards *Picea abies*, while during saprophytic growth it degrades plant litter [14,15]. It is genetically amenable, and can be grown in culture under axenic conditions with its natural host [14]. While fast-growing ectomycorrhizal fungi have been used widely to study symbiosis, late-stage mycorrhizal fungi have rarely been used [15,16]. However, since they are replacing fast-growing generalist mycorrhizal partners in later successional stages, the communication system, including volatiles, may be more specific with these [16]. Hence, *T. vaccinum* was used in this study as a model for late-stage ectomycorrhizal fungi. The production of multiple compounds, including volatiles and phytohormones, has been shown [16,17].

The synthesis of isoprenoids can proceed via the methyl erythritol 4-phosphate (MEP/DOXP) pathway, starting from farnesol-pyrophosphate [18,19], or, as known for fungi, using the mevalonate pathway [5]. Isotope labeling has shown the synthesis of geosmin to occur via the mevalonate pathway for the basidiomycete *T. vaccinum* [16]. A sesquiterpene cyclase then generates a cationic intermediate that undergoes a series of transformations, until a stable product is formed that consists of several products with different skeletal scaffolds after rearrangement [5,13]. In the case of melleolide biosynthesis, Δ^6^-protoilludene is the first stable product [20]. The ecological role of fungal sesquiterpenes may include acting as pheromones, allomones, or kairomones [21]. In ectomycorrhizae, thujopsene, a sesquiterpene from *Laccaria bicolor*, was found to enhance lateral root elongation and root hair prolongation in *Populus*, and to promote superoxide anion radical formation in the meristemic zone of the root tip [21,22]. Dehydroaromadendrene, β-cubebene, and longicyclene have been reported to promote the symbiosis between *Tuber borchii* and *Tilia americana* without contact, at the same time eliciting hyphal elongation [23].

Coding for a terpene synthase, the production of geosmin by the ectomycorrhizal *T. vaccinum* has been found to occur, depending on the activity of *ges1*. The knockdown of this gene reduced the amount of geosmin in the headspace of the fungus [24]. In this previous study, we focused on the major products, while in this study we attempted to learn more about the full spectrum of volatiles involved in the mycorrhization process.

The sampling of below-ground headspace, excluding the contamination by above-ground, tree-needle-derived compounds, requires static sampling with microfibers for solid-phase microextraction (SPME) [23,25,26]. We adapted the technique for mycorrhizal interactions to compare axenic versus ectomycorrhizal VOC representation in the headspace. We then identified a comprehensive set of potential biosynthetic sesquiterpene synthase genes by analyzing differential expression in mycorrhiza. With this approach, we identified γ-protoilludene (**2**) and its putative protoilludene synthase, *pie1*, as one of the major biosynthetic pathways during *T. vaccinum* ectomycorrhizal interaction with its host, *P. abies*.

## 2. Materials and Methods

### 2.1. Cultivation of T. vaccinum and Germination of Seeds of P. abies

*T. vaccinum* GK6514 (SF004731, Jena Microbial Resource Collection, Jena, Germany) was cultivated on an agar medium (Modified Melin Norkrans MMNb) [27] at 23 °C for 4 weeks. *Picea abies* (Karst.) seeds (Landesforst Mecklenburg-Vorpommern, Germany) were immersed in tap water over night, surface-sterilized using 30% H_2_O_2_ for 60 min, washed, and germinated [28].

### 2.2. Sampling of Below-Ground Volatiles

**Figure 1:**
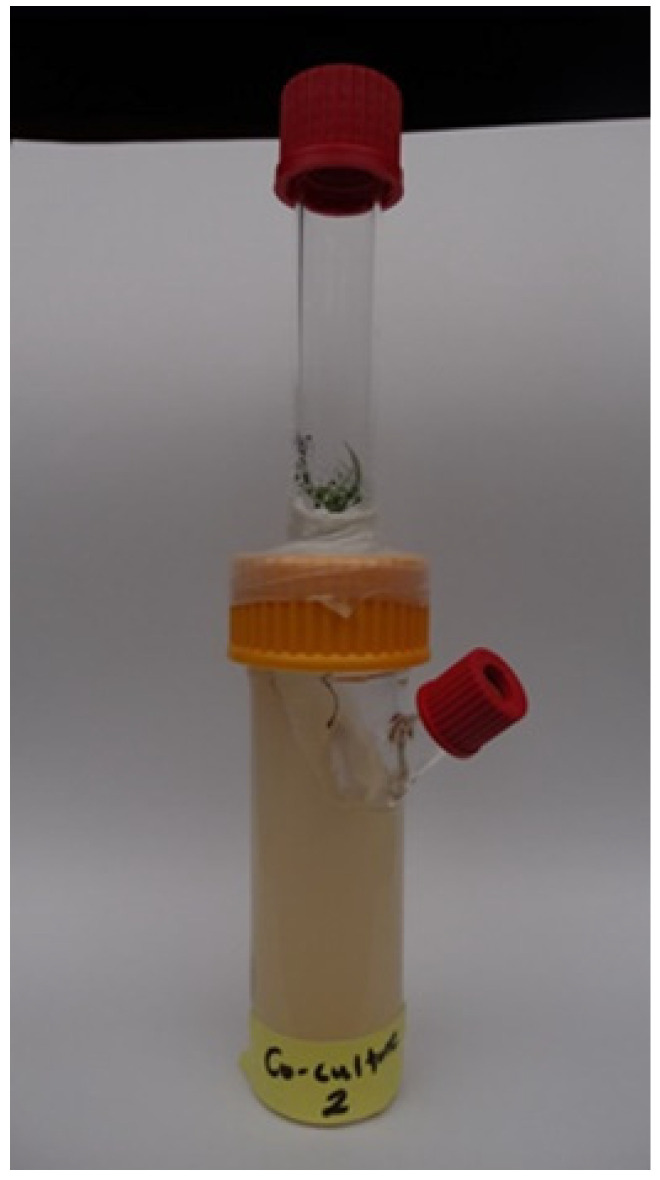
Experimental set up for the sampling of root volatile organic compounds. The green tree parts are visible and separated from the below-surface root fungus system, where sampling was made possible through the inlet Teflon cap visible at the right.

A solid MMNa medium (MMNb with 0.5 g/L (NH_4_)_2_HPO_4_, 2 g/L D-glucose, and without malt extract) [27] was used for axenic *T. vaccinum* cultures and *P. abies–T. vaccinum* co-cultivation in a climate chamber with a day/night cycle of 12 h at 23/17 °C at 80% humidity. For separating below-ground from above-ground headspaces, the two chambers were separated by a Teflon cork through which the stem could be passed. The stem was then sealed with agar to prevent the passage of volatiles (Figure 1). The fungus was then inoculated into the lower chamber to allow contact with the root. The volatiles were sampled via inlet Teflon caps using SPME extraction (Supelco, Bellafonte, PA, USA) coated with three different stationary phases (polydimethylsiloxane 100 µm, polydimethylsiloxane/divinylbenzene 65 µm, and divinylbenzene/carboxene/polydimethylsiloxane 50/30 µm). The fibers were conditioned according to the manufacturer instructions. To prepare the fibers for sampling, they were cleaned by inserting them into the inlet of the gas chromatograph (GC) for 5 min at 250 °C.

### 2.3. GC–MS Analysis of Volatiles

The headspace VOCs were sampled regularly for 48 h over a period of 6 weeks using SPME fibers. Individual fibers were used for re-sampling one biological replicate, as SPME fibers may differ in their loading capacity. The SPME fibers were then applied to desorption in the GC-inlet, and the compounds analyzed by gas chromatography–mass spectrometry (GC–MS; Trace 1310 GC and ISQ LT MS detector, Thermo Fisher Scientific GmbH, Dreieich, Germany), equipped with a ZB5 column (30 m × 0.25 mm × 0.25 µm) with a 10 m Guardian End (Phenomenex, Aschaffenburg, Germany). Measurements were executed in electron impact (EI) mode with 70 eV (*m/z* 33 to 450) at 1.5 mL/min helium. The GC injector (split ratio 1:20), transfer line, and ion source were set at 230 °C, 280 °C, and 250 °C, respectively. The volatiles were eluted under programmed conditions from 40 °C (hold for 2 min), followed by a 10 °C/min increase to 230 °C (cleaning step 50 °C/min increase to 300 °C) [29].

### 2.4. Identification of Sesquiterpenes of T. vaccinum

The mixed n-alkanes C8 through C20 in n-hexane (Sigma-Aldrich, Saint Louis, MI, USA) were measured under the same conditions as the samples. Retention indices (RI) were calculated, and all compounds were preliminary identified by RI. Wherever possible, the identity was confirmed by direct comparison to authentic references. In addition, their mass spectra (MS) and RIs were compared to mass spectral libraries (NIST/EPA/NIH; Wiley, Hoboken, NJ, USA), Massfinder MS 4 and 4 with the Adams collection RI data, in combination with mass spectral library (NIST/EPA/NIH) 53 MS search. RIs deviating more than ±2 from the authentic references and ±5 from the database were regarded as mismatches, and not considered [7,29]. Spectra of the fungal volatile α-barbatene (**6**) were obtained using an authentic standard (kindly provided by Prof. Stefan von Reuss, Neuchatel, Switzerland), while Δ^6^-protoilludene (**2**) was identified using headspace volatiles from *Ophiostoma picea* (provided by Dineshkumar Kandasamy, MPI Chemical Ecology, Jena, Germany).

### 2.5. Homology Searches and Phylogenetic Tree Reconstruction

Sequences of fungal sesquiterpene synthases of *Antrodia cinnamomea*, *Armillaria gallica*, *Boreostereum vibrans*, *Coprinopsis cinerea*, *Coniophora puteana*, *Fomitopsis pinicola*, *Lignosus rhinoceros*, *Omphalotus olearius,* and *Stereum hirsutum* [30] were employed for phylogenetic tree reconstruction. Sesquiterpene synthase genes of *Coprinopsis cinerea* (Cop1 through Cop6) were used in a Blast search (https://blast.ncbi.nlm.nih.gov/Blast.cgi, accessed on 1 March 2022) against the genome of *T. vaccinum* (available at genome.jgi.doe.gov/portal, accessed on 1 March 2022). Sequence alignments were computed using ClustalW [31], and alignment and phylogenetic reconstructions were performed as implemented on GenomeNet (https://www.genome.jp/tools/ete (accessed on 1 March 2022)) [32]. The maximum likelihood tree was inferred using PhyML (v20160115) [33]. Branch supports were computed out of 100 bootstrapped trees. The phylogenetic tree dataset was thereafter designed using iTOL (www.itol.embl.de, accessed on 23 January 2022).

### 2.6. Expression Analyses by qRT-PCR for Putative Sesquiterpene Synthase Genes

The influence of co-cultivation on putative sesquiterpene synthase gene expression was analyzed in 3-week-old co-cultures compared to axenic *T. vaccinum* by qPCR. Total RNA was extracted (RNeasy Plant Mini Kit, Qiagen, Hilden, Germany) [34], cDNA was synthesized using QuantiTect Reverse Transcription Kit (Qiagen, Hilden, Germany), and changes in gene expression for nine candidate genes (g334, g1826, g1880, g2958, g5920/*ges1*, g4529, g7856, g6053 and g10283) were monitored. For reference, *cis1*, *act1*, and *tef1* were used with three technical replicates (for primers used, Table 1). Quantification was carried out using qPCR (Cepheid Thermocycler, Sunnyvale, CA, USA) with Maxima SYBR Green 2x Master Mix (Thermo Fisher Scientific, Waltham, MA, USA). Primer efficiencies were calculated using a dilution series, and expression ratios were normalized as described [35]. Statistical significance was checked using a Student’s *t*-test.

## 3. Results

### 3.1. Sesquiterpenes Produced by Axenically Grown T. vaccinum

**Figure 2:**
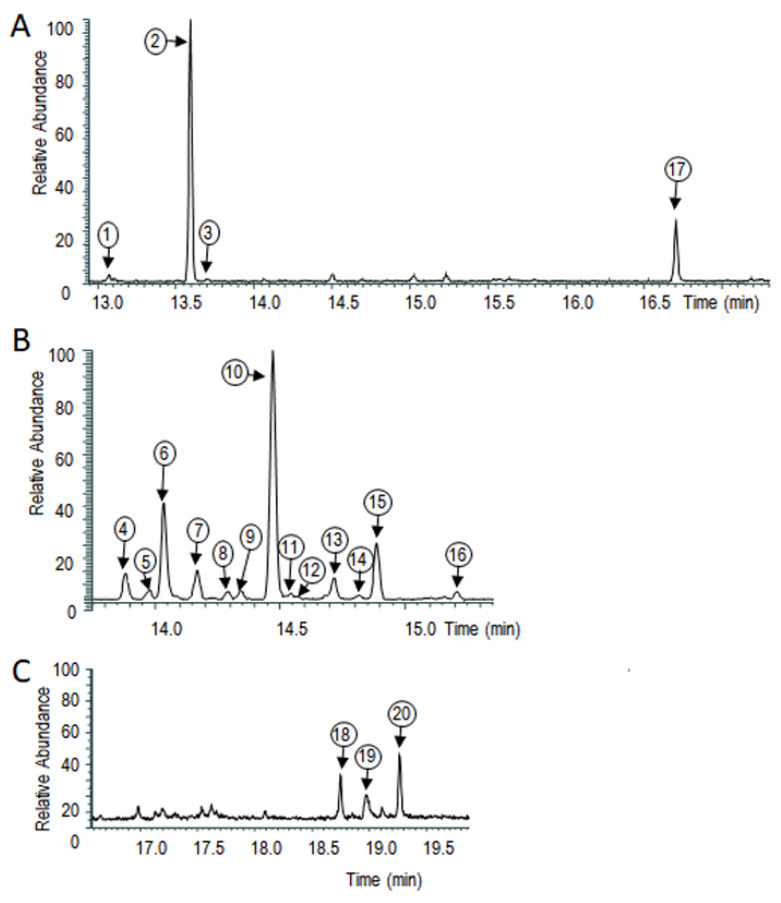
Sesquiterpenes formed by axenic *T. vaccinum.* (**A**) The major peaks are visible with the entire spectrum. (**B**) Enlarged view allows us to differentiate peaks number 4 through 16. (**C**) Enlarged view of the peaks eluting at higher retention times, numbers 18 through 20. The peaks were numbered consecutively; for each peak a number is given in a small circle above the peak to identify the compound for further analyses. The retention times of the 20 peaks were used for identification with authentic standards, and by mass spectrometry (see Supplementary Table S1). The peak numbers (**2**) (Δ^6^-protoilludene), (**3**) (sativene), (**4**) (isocaryophyllene), (**5**) (β-cedrene), (**6**) (α-barbatene), (**7**) (β-copaene), (**8**) (thujopsene), (**10**) (β-barbatene), (**13**) (4-epi-α-acoradiene), (**15**) (chamigrene), and (**16**) (α-cupraene) could be identified, while peaks (**1**), (**9**), (**11**), (**12**), (**14**) and (**17**)–(**20**) did not yield an identification (for mass spectra, see Supplementary Figure S1).

Upon cultivation, *T. vaccinum* produced more than 20 detectable VOCs (Figure 2). From those, Δ^6^-protoilludene (**2**; the numbers refer to each compound identified in Figure 2), sativene (**3**), isocaryophyllene (**4**), β-cedrene (**5**), α-barbatene (**6**), α-copaene (**7**), thujopsene (**8**), β-barbatene (**10**), 4-epi-α-acoradiene (**13**), chamigrene (**15**), and α-cupranene (**16**) could be identified (Supplementary Table S1; for mass spectrometry data see Supplementary Figure S1). Among the unidentified sesquiterpenes, one of the major compounds was tentatively assigned as a sesquiterpene ketone. The three major components of the volatilome of *T. vaccinum* were the VOCs Δ^6^-protoilludene (**2**) eluting at 13.57 ± 0.03 min, β-barbatene (10; 14.47 ± 0.03 min), and the unidentified sesquiterpene ketone (17; 16.67 ± 0.03 min), with β-barbatene appearing as the dominating peak. For the major compounds, Δ^6^-protoilludene (**2**), β-barbatene (**10**), and the putative sesquiterpene ketone (**17**), a role in signaling during mycorrhization was checked.

### 3.2. The Volatilome of T. vaccimum Is Modified by Mycorrhization

**Figure 3:**
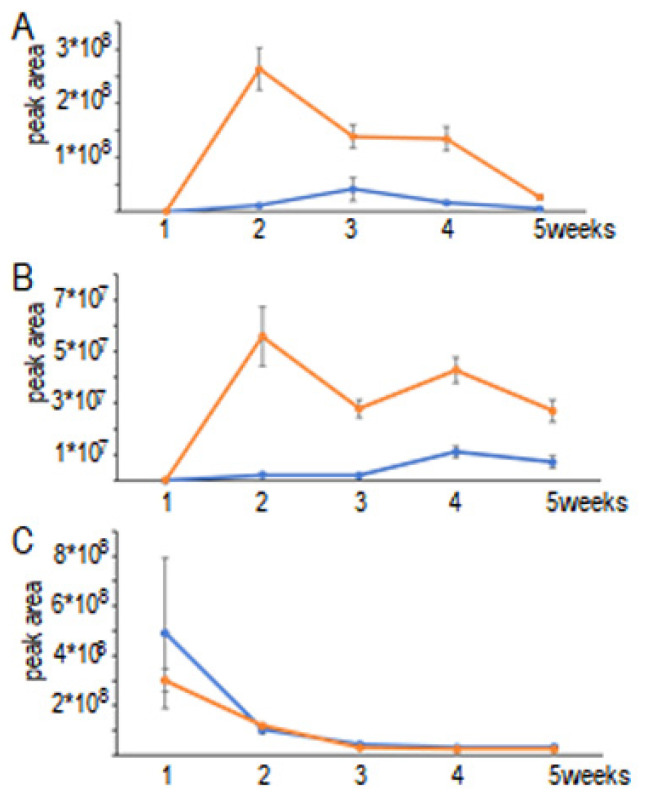
Dynamics of the production of major sesquiterpenes. Quantification in axenic (blue) and ectomycorrhizal (orange) conditions for (**2**) Δ^6^-protoilludene (**A**), (10) β-barbatene (**B**), and the (**17**) sesquiterpene ketone (**C**) are given; *n* = 5.

The volatiles produced were monitored over six weeks during growth under mycorrhizal conditions. With this approach, the peak areas of the three major components could be correlated with their production rates during the co-cultivation of *T. vaccinum* with its host, *P. abies*. The minor sesquiterpenes were below detection limits at different time points. However, the major compounds were tractable throughout, showing distinct dynamics for Δ^6^-protoilludene (**2**), α-(**6**) and β-barbatene (**10**), and the putative sesquiterpene ketone (**17**). A decrease in intensity was observed progressively over incubation times for ß-barbatene (**10**). Both Δ^6^-protoilludene (**2**) and the major unidentified sesquiterpene ketone (**17**) appeared at increasing amounts in co-culture with the host, *P. abies*, where they appeared one week earlier compared to the volatilome from axenic conditions (Figure 3; Supplementary Figure S2).

### 3.3. Multiple Genes for Sesquiterpene Synthases Are Present in the T. vaccinum Genome

**Figure 4:**
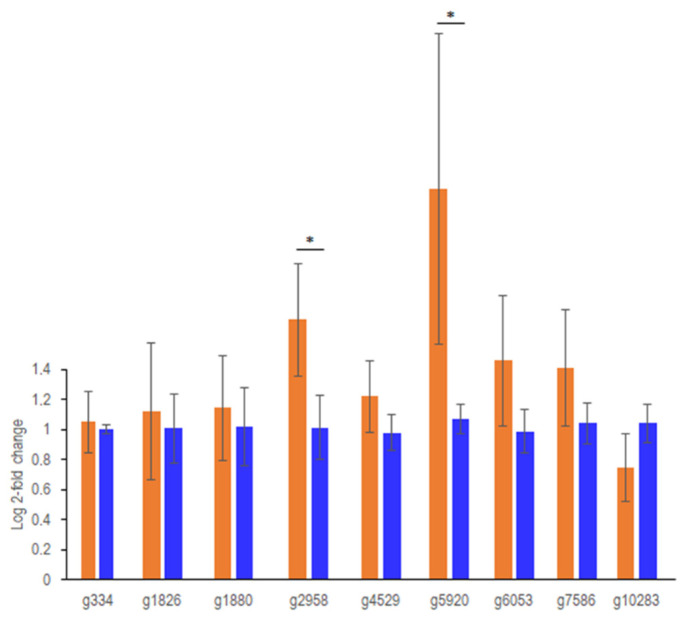
Expression of sesquiterpene synthases with log2-fold changes measured by qRT-PCR in *T. vaccinum*. Axenic (blue) and mycorrhiza with *P. abies* (orange) were measured. The gene identifiers for the nine genes, coding for putative sesquiterpene synthases, are given; *n* = 3. Statistical significance is indicated with a star.

A predictive framework based on the known *C. cinerea* sesquiterpene synthases Cop1 through Cop6 was employed to identify nine genes encoding putative sesquiterpene synthases from the *T. vaccinum* genome at 30 to 70% similarity, including the already identified geosmin synthase, *ges1* [16]. Since we were interested in mycorrhiza-specific VOCs, we tested the differential expression of these nine genes in mycorrhiza by qRT-PCR (Figure 4). While most genes showed similar expression under both conditions, one (gene identifier g5920; *ges1*, see [24]) appeared with six-fold higher amounts, and a second gene (g2958) with a four-fold increase under co-cultivation conditions. Most strikingly, under the cultivation conditions used here to identify a higher diversity of sesquiterpenes, the previously observed geosmin was not produced.

**Figure 5:**
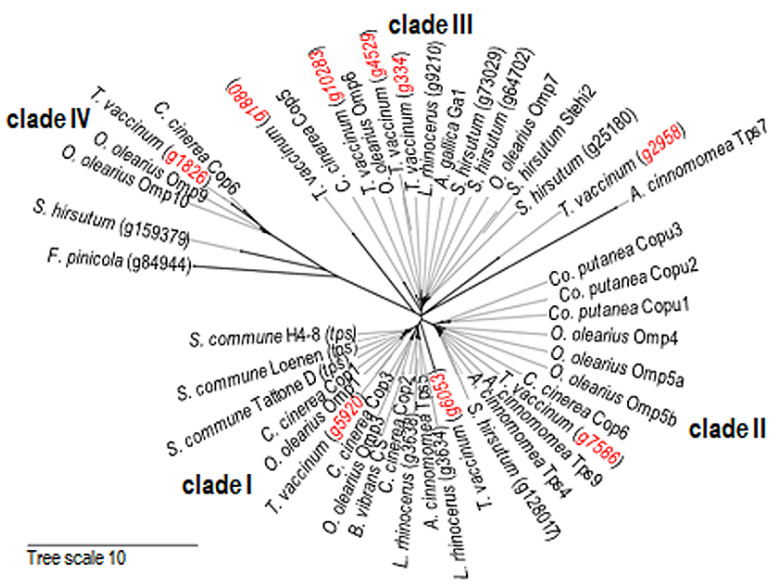
Phylogenetic clustering of putative basidiomycete sesquiterpene synthases. The protein sequences identified from the *T. vaccinum* genome are labeled in red; conceptually translated protein sequences of other basidiomycetes are from *Antrodia cinnamomea*, *Armillaria gallica*, *Boreostereum vibrans*, *Coprinopsis cinerea*, *Coniophora puteana*, *Fomitopsis pinicola*, *Lignosus rhinoceros*, *Omphalotus olearius*, *Stereum hirsutum*, and *Schizophyllum commune*; gene identifiers are given in brackets if no protein names are available.

Phylogenetic tree reconstruction using basidiomycete sesquiterpene synthase genes [5,30] placed the geosmin synthase *ges1* into a cluster of functional clade I with enzymes known to catalyze the 1,10 cyclization of *E*,*E*-farnesyl pyrophosphate (Figure 5). Another gene, g7586, was associated with clade II enzymes catalyzing the 1,10 cyclization of 3*R*-nerolidyl pyrophosphate. Gene g2958 (as well as g6053) is also statistically significantly up-regulated in mycorrhiza. These gene products were placed in the tree to cluster at the base of clade III proteins that 1,11-cyclize *E*,*E*-farnesyl pyrophosphate. In addition to these two basal lineages, products of g334, g4529, and g10283 were found together with other characterized Δ^6^-protoilludene synthases. The members of clade IV, coding for known barbatene synthases, and catalyzing the 1,6 cyclization of 3*R/S*-nerolidyl pyrophosphate, included g1880 and g1826. With this, we could predict the up-regulated g2958, now referred to as *pie1*, into a group of genes known to encode Δ^6^-protoilludene synthases.

## 4. Discussion

In order to identify the sesquiterpenes produced by the ectomycorrhizal fungus *T. vaccinum*, a sampling device was used that allowed for both axenic culture or mycorrhiza volatile extraction without interference from above-ground tree VOCs. The ectomycorrhizal basidiomycete *T. vaccinum* was observed without and with its specific partner, Norway spruce (*P. abies*). Basidiomycete sesquiterpenes Δ^6^-protoilludene (**2**), α-(**6**) or β-barbatene (**10**), have been observed previously, while the oxygenated sesquiterpene, putatively identified here as a ketone (**17**), has not yet been reported [36]. Early in the axenic culture of *T. vaccinum*, β-barbatene (**10**) was seen as the most abundant compound, in concordance with sesquiterpenes appearing during mycelial growth in other ectomycorrhizal basidiomycetes [37]. The decline in barbatene concentrations observed after new growth had been established might hint at a reaction to mechanical damage incurred during mycelial plug cutting and transfer, as it has been shown for the typical mushroom odor compounds 1-octen-3-ol and 3-octanone, which increase by up to 10-fold after mushroom cutting [38]. In *Fomitopsis,* oct-1-ene and octan-3-one, as well as β-barbatene (**10**), responded to the chopping of one-week old fruiting bodies, but also under natural conditions at the sporulation stage [39]. Since *T. vaccinum* does not form fruiting bodies under co-cultivation conditions, a response to mechanical stress seems the likely reason for its increased production after the transfer of mycelial plugs.

While VOCs have so far mostly been recorded from mushrooms, and hence during sporulation [13,20,21,40], *T. vaccinum* produced Δ^6^-protoilludene during mycelial, vegetative growth. Protoilludene was long thought to be a biomarker for basidiomycetes, until it was identified from other fungi as well, and it has also been associated with bark beetle attack [41,42,43]. In our study, Δ^6^-protoilludene (**2**) production was highly induced under mycorrhizal conditions, a dynamic that is shared with a potential oxidation product, a sesquiterpene ketone (**17**).

In contrast to ß-barbatene (**10**), the production of Δ^6^-protoilludene (**2**), after an initial increase, steadily declines over time in mycorrhiza, as is the case for the putative sesquiterpene ketone (**17**), suggesting that both compounds may be produced from a sesquiterpene by subsequent oxidation involving a cytochrome P450 enzyme. The putative ketone compound has a mass of *m/z* 218.18, indicative of the presence of a scaffold containing a single oxygen atom. Most oxygenations in sesquiterpenes are catalyzed by specific cytochrome P450 monooxygenases, often encoded adjacent to the respective sesquiterpene synthase gene [44]. We hypothesize that the oxygenated sesquiterpene may be a derivative of protoilludene. Both Δ^6^-protoilludene (**2**) and the oxygenated sesquiterpene (**17**) formed in increased amounts in *T. vaccinum* under co-cultivation with its spruce host, which may suggest that these are host communication molecules.

Most basidiomycetes possess inherent abilities to produce sesquiterpenes with varied and unpredictable scaffolds [1,7,45]. This is linked to promiscuity in sesquiterpene synthases, and associated with linked genes coding for modifying enzymes. Two sesquiterpene synthases of *Stereum hirsutum* (gene identifiers 64702, 73029), both belonging to clade III, produced germacrene A-derived products via 1,10 cyclization, which is found with members of clade I, while Δ^6^-protoilludene (**2**) is usually seen to originate from 1,11 cyclization by members of clade III. Thus, some sesquiterpenes can be produced through multiple mechanistic pathways. Additionally, members of clades I and II sesquiterpene synthases are well known for their non-specific product formation [7]. The sequence ambiguity that characterizes most sesquiterpenes of fungi challenges the in silico functional characterization.

We identified the genes for several sesquiterpene synthases of 30–70% similarity to the *C. cinerea* enzymes Cop1–6. While most genes were expressed under axenic and co-cultivation conditions alike, *ges1* (g5920), as well as a second gene (g2958), showed a six- and four-fold upregulation, respectively, in mycorrhiza. While *ges1* was clustering with Cop1, Cop2, and Cop3 in clade I, known to be involved in germacrene A production, the newly identified, up-regulated putative sesquiterpene synthase clusters at the base of clade III. This prospective protoilludene synthase, *pie1* (g2958), shares a good clustering score with the well-characterized protoilludene synthase from *Armillaria gallica*, using a sequence similarity network construction [46,47].

## 5. Conclusions

This study identified sesquiterpenes and their biosynthetic enzymes from the ectomycorrhizal fungus *T. vaccinum.* The applied sampling allowed for the identification of minor compounds of the volatilome that had not been detected previously. The sesquiterpenes Δ^6^-protoilludene (**2**) and an oxygenated (potentially derivative) sesquiterpene ketone (**17**) specifically increased in amount in mycorrhiza, as was the transcript for the prospective synthase *pie1*. With this work, we identified putative signaling compounds and an accordingly regulated gene. This will pave the way to unravel host communication via VOCs in the ectomycorrhizal root system, both during establishment and in mycorrhizal morphotypes.

## Figures and Tables

**Table 1:** Oligonucleotide primers used in this study.

Gene	Forward Primer Sequence	Reverse Primer Sequence
g334	CCCAGTGCATCCATGTTGTA	CTGGCTGTCAATCACACACT
g1826	GCAACCCATCGCGGATATAA	ATCAGGAAGGTGCGCATAAG
g1880	TCACCACCATGTTCGACTTT	CCTGTCGATCACGCACATAC
g2958	CGACAAACCCGATACTCCAAATA	GGTCTGTAGAGAAGCATGTACC
g4529	AGGAGGTGGTTGCTACTTTG	GGTAGCGCGACAGTAAGTATAG
g5920	GGCACCCAGCGAAGATTTAT	CACGACGAAGAGCGATGTATG
g6053	GTTGTGGCGTCTCGGATATT	GGCTCGACGTCGTTGTATT
g7586	TCGACTGCTGCCCAATAAAT	GTGCTGTTCGACTTTGCTTTAG
g10283	GATGGATTGGGATACTGGGTTC	GAGCGTAACCCAACGAGATT
*act1*	ACAACCATGTTCCCCGGTATCT	TTCGCTCAGGAGGAGCAACAAT
*cis1*	CAAATTCGTGCCGAGCATGG	ACCCGTCCCAGATGAGAGCA
*tef1*	GGCAACTTATTGTTGCTGTGAACAA	GACCTTCTTGATAAAGTTGGAGGTT

## Data Availability

Not applicable.

## Supplementary Materials

The following supporting information can be downloaded at: https://www.mdpi.com/article/10.3390/jof8060555/s1: Table S1: Characterization of 20 volatiles produced by *T. vaccinum* in axenic culture; Figure S1: Mass spectra with relative abundance for the sesquiterpenes; Figure S2: Dynamics of the production of major sesquiterpenes.
