# Supplementary material for: Ectomycorrhizal Influence on the Dynamics of Sesquiterpene Release by Tricholoma vaccinum
Source: J Fungi (Basel). 2022 May 24;8(6):555. doi: 10.3390/jof8060555 (PMC9224709; doi:10.3390/jof8060555)
Supplement: Supplementary file 1 [file jof-08-00555-s001.zip › jof-1716541-supplementary.pdf]

Supplemental material

Supplemental Table S1: Characterization of 20 volatiles produced by *T. vaccinum* in axenic culture for compound identification.

S/N	Retention time	Retention index (RI)	*m/z*	Sesquiterpene(oid)	Mode of identification
1	13.07	1348	204.19	no match	RI
2	13.52	1384	204.19	Δ^6-protoilludene	RI vs. standard
3	13.69	1395	204.18	sativene	RI, spectra
4	13.88	1410	204.18	isocaryophyllene	RI, spectra (NIST)
5	13.97	1417	204.21	ß-cedrene	RI
6	14.03	1421	204.2	α-barbatene	RI vs. standard
7	14.16	1432	204.18	β-copaene	RI, spectra (NIST)
8	14.28	1442	204.19	thujopsene	RI, spectra (NIST)
9	14.33	1446	204.15	no match	RI
10	14.47	1456	204.18	ß-barbatene	RI
11	14.53	1462	204.23	no match	
12	14.68	1473	204.17	no match	RI
13	14.71	1475	204.21	4-epi-α-acoradiene	RI
14	14.81	1483	204.19	no match	RI
15	14.88	1489	204.19	chamigrene	Spectra (NIST)
16	15.2	1515	202.17	α-cupraene	RI
17	16.68	1643	218.18	no match	RI
18	18.75	1832		no match	
19	18.98	1854	218.16	no match	
20	19.27	1882	234.15	no match	RI

NIST, mass spectral library

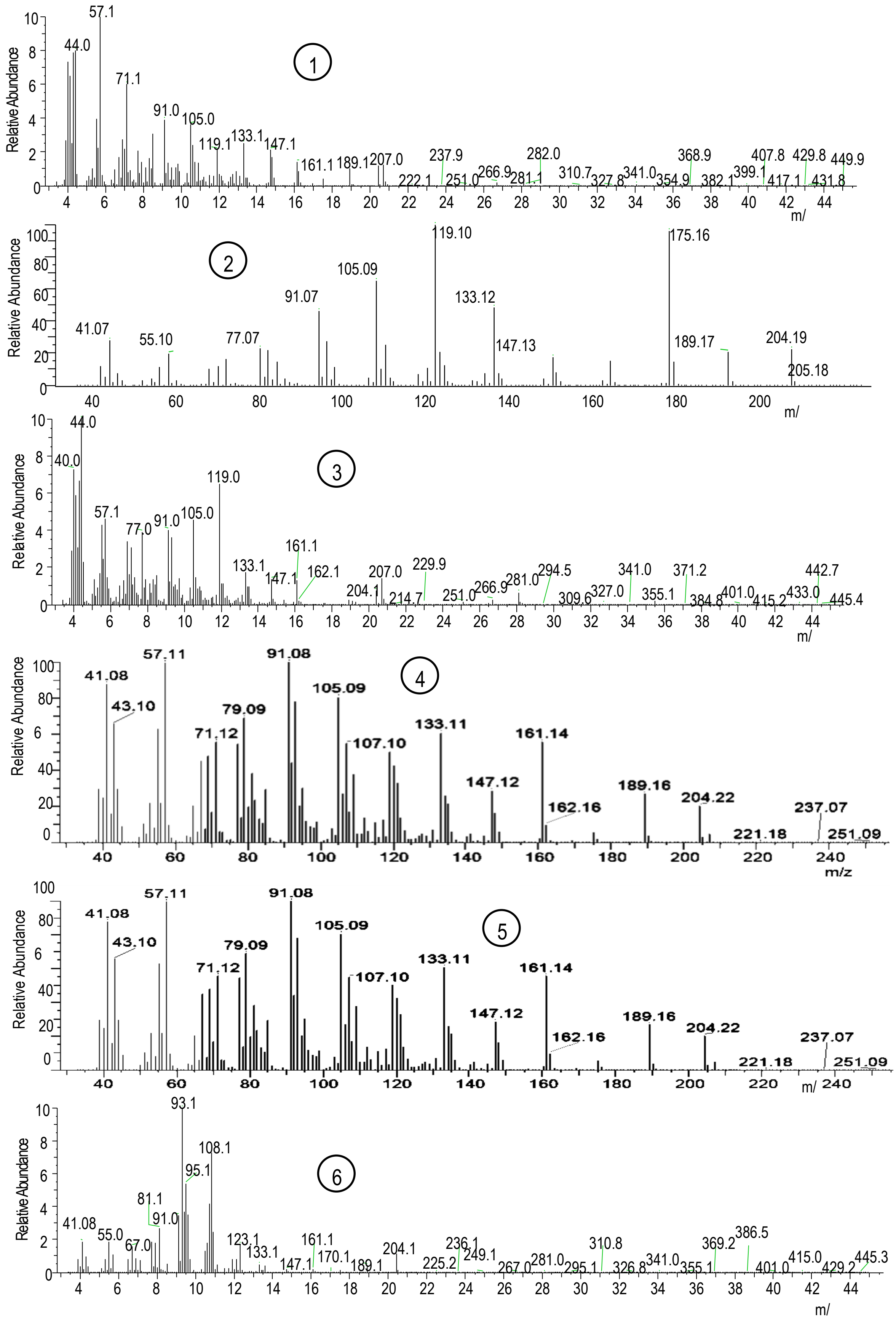

1
Relative Abundance
57.1
44.0
71.1
91.0
105.0
119.1
133.1
147.1
161.1
189.1
207.0
222.1
237.9
251.0
266.9
281.1
282.0
310.7
327.8
341.0
354.9
368.9
382.1
399.1
407.8
417.1
429.8
431.8
449.9
m/
2
Relative Abundance
41.07
55.10
77.07
91.07
105.09
119.10
133.12
147.13
175.16
189.17
204.19
205.18
m/
3
Relative Abundance
44.0
40.0
57.1
77.0
91.0
105.0
119.0
133.1
147.1
161.1
162.1
204.1
207.0
214.7
229.9
251.0
266.9
281.0
294.5
309.6
327.0
341.0
355.1
371.2
384.8
401.0
415.2
433.0
442.7
445.4
m/
4
Relative Abundance
41.08
43.10
57.11
71.12
79.09
91.08
105.09
107.10
133.11
147.12
161.14
162.16
189.16
204.22
221.18
237.07
251.09
m/z
5
Relative Abundance
41.08
43.10
57.11
71.12
79.09
91.08
105.09
107.10
133.11
147.12
161.14
162.16
189.16
204.22
221.18
237.07
251.09
m/
6
Relative Abundance
93.1
108.1
95.1
81.1
91.0
41.08
55.0
67.0
123.1
133.1
147.1
161.1
170.1
189.1
204.1
225.2
236.1
249.1
267.0
281.0
295.1
310.8
326.8
341.0
355.1
369.2
386.5
401.0
415.0
429.2
445.3
m/

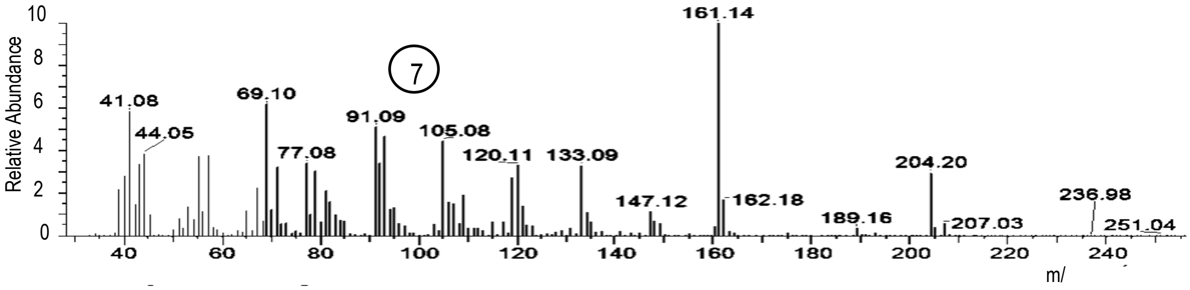
7
Relative Abundance
41.08
44.05
69.10
77.08
91.09
105.08
120.11
133.09
147.12
161.14
162.18
189.16
204.20
207.03
236.98
251.04
m/

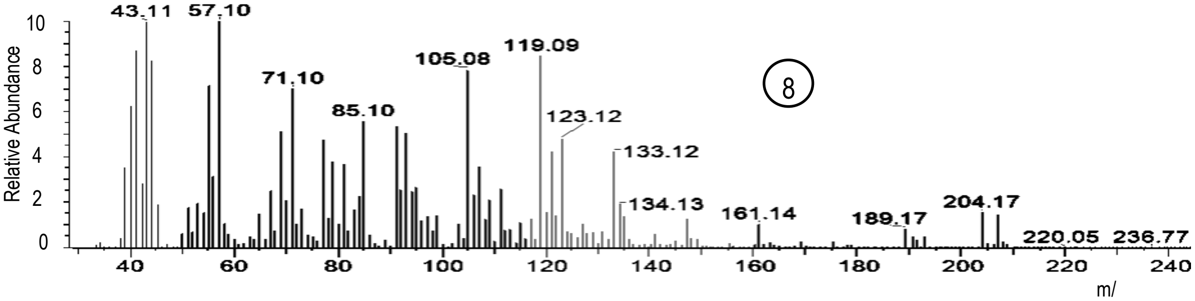
8
Relative Abundance
43.11
57.10
71.10
85.10
105.08
119.09
123.12
133.12
134.13
161.14
189.17
204.17
220.05
236.77
m/

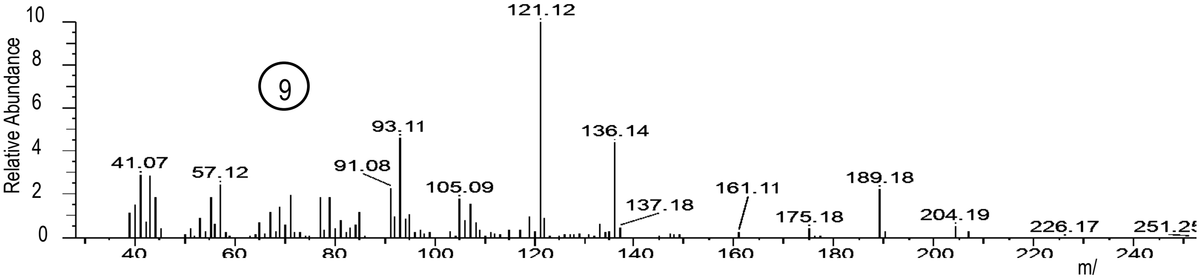
9
Relative Abundance
41.07
57.12
91.08
93.11
105.09
121.12
136.14
137.18
161.11
175.18
189.18
204.19
226.17
m/

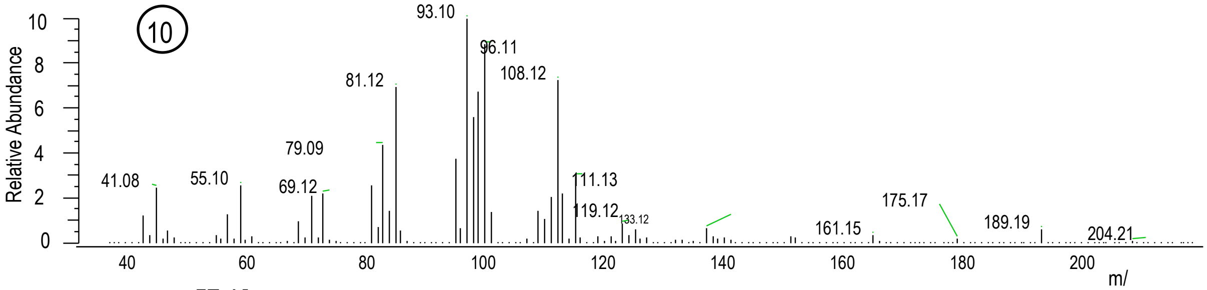
10
Relative Abundance
41.08
55.10
69.12
79.09
81.12
93.10
96.11
108.12
111.13
119.12
133.12
161.15
175.17
189.19
204.21
m/

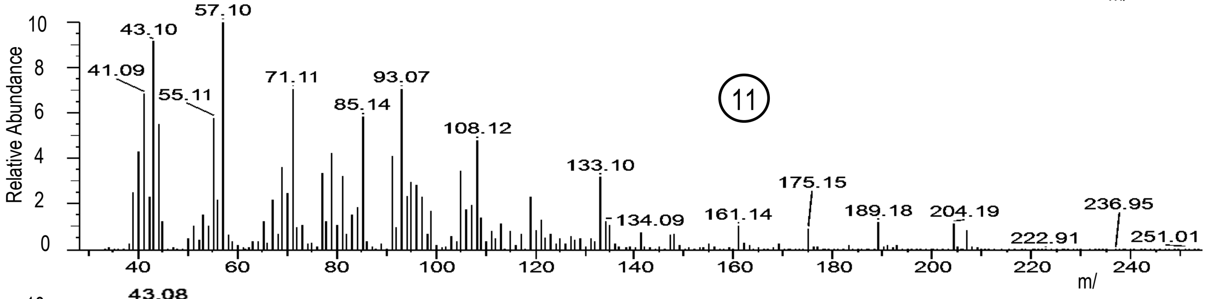
11
Relative Abundance
41.09
43.10
55.11
57.10
71.11
85.14
93.07
108.12
133.10
134.09
161.14
175.15
189.18
204.19
222.91
236.95
251.01
m/

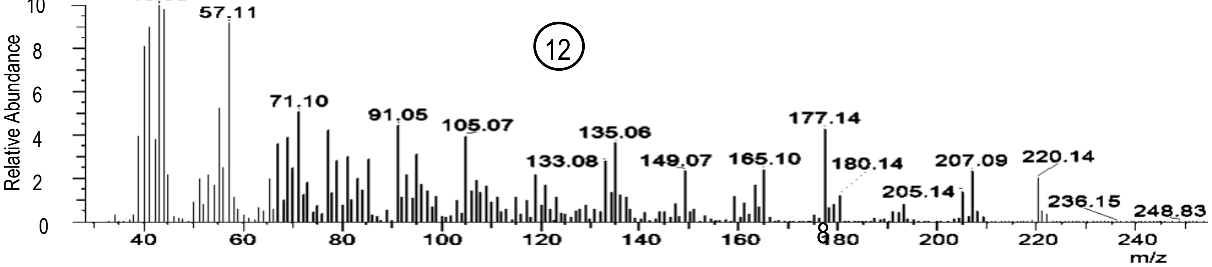
12
Relative Abundance
43.08
57.11
71.10
91.05
105.07
133.08
135.06
149.07
165.10
177.14
180.14
205.14
207.09
220.14
236.15
248.83
m/z

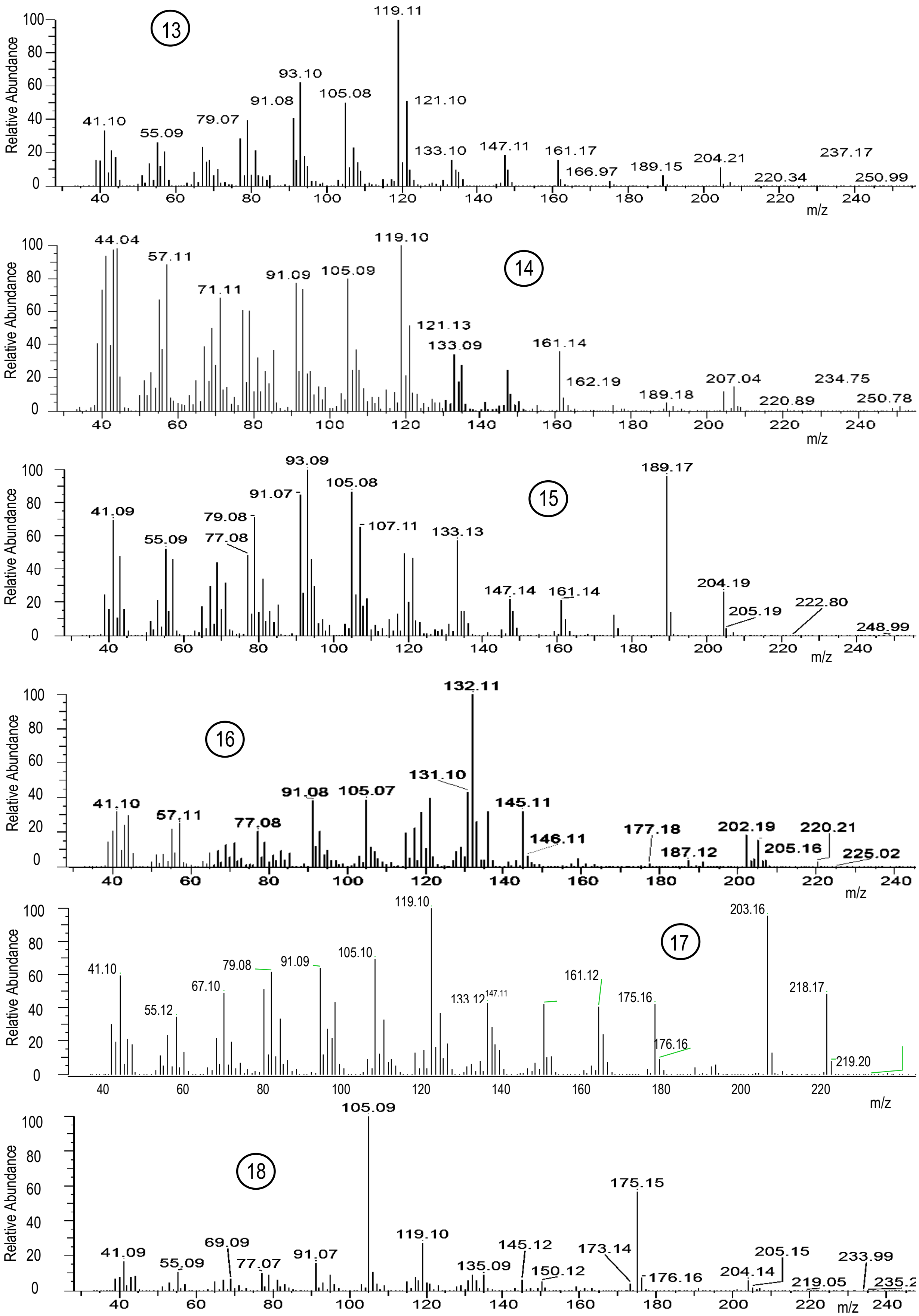
13
Relative Abundance
m/z
119.11
93.10
105.08
121.10
91.08
41.10
79.07
55.09
133.10
147.11
161.17
166.97
189.15
204.21
220.34
237.17
250.99
14
44.04
57.11
119.10
91.09
105.09
71.11
121.13
133.09
161.14
162.19
189.18
207.04
220.89
234.75
250.78
15
93.09
189.17
91.07
105.08
41.09
79.08
107.11
133.13
55.09
77.08
147.14
161.14
204.19
205.19
222.80
248.99
16
132.11
131.10
41.10
91.08
105.07
145.11
57.11
77.08
146.11
177.18
187.12
202.19
205.16
220.21
225.02
17
119.10
203.16
41.10
79.08
91.09
105.10
55.12
67.10
133.12
147.11
161.12
175.16
176.16
218.17
219.20
18
105.09
175.15
41.09
55.09
69.09
77.07
91.07
119.10
135.09
145.12
150.12
173.14
176.16
204.14
205.15
219.05
233.99
235.2

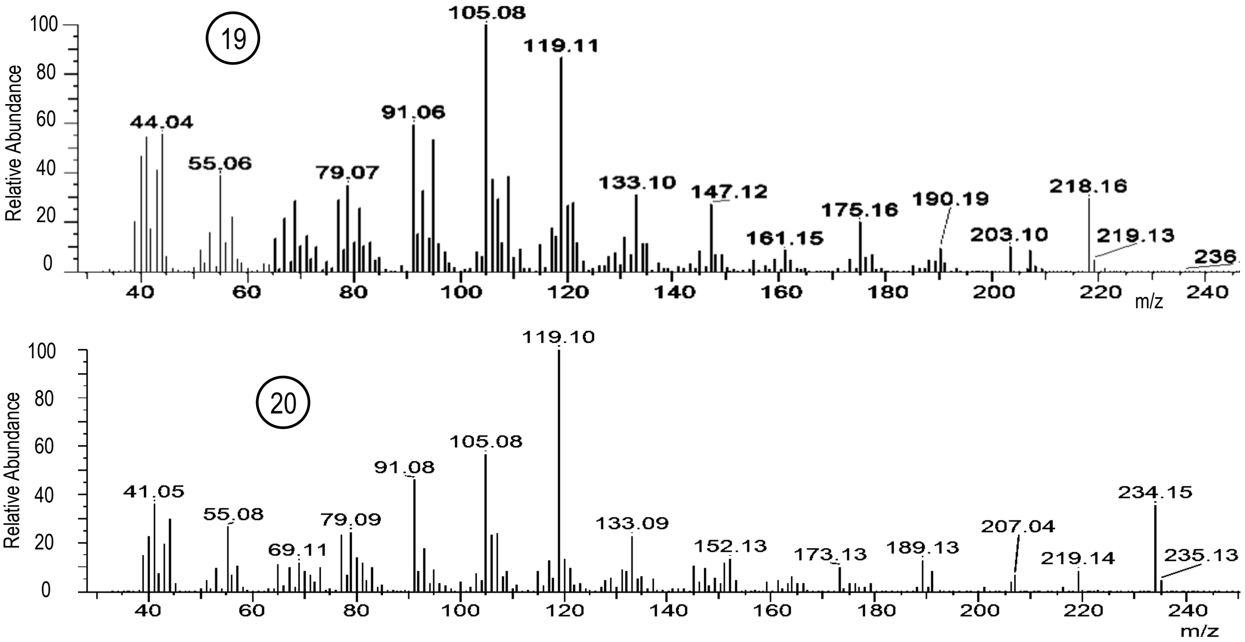

Supplemental Figure S1: Mass spectra (*m/z*) with relative abundance for the sesquiterpenes numbered Fig. 2. The sesquiterpenes 1 through 20 produced by *T. vaccinum* were identified by retention index and mass spectra and are listed in suppl. Tab. S1.

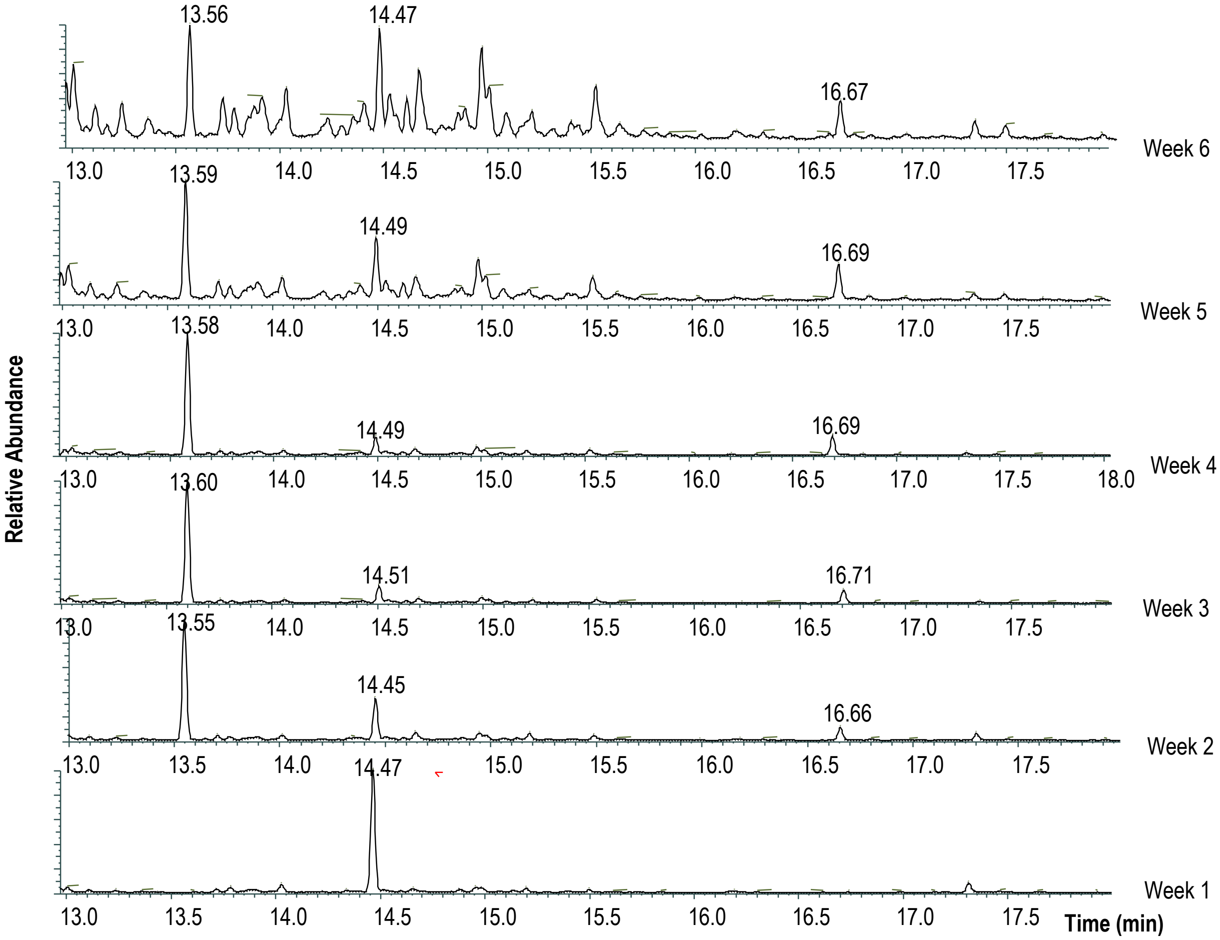

Supplemental Figure S2: Dynamics of the production of major sesquiterpenes. Samples were taken at week 1 through 6 and chromatograms were taken to show the appearance of peaks for the major sesquiterpenes in co-culture.
